# Endovascular and Clinical Outcomes of Vertebrobasilar Intracranial Atherosclerosis-Related Large Vessel Occlusion

**DOI:** 10.3389/fneur.2019.00215

**Published:** 2019-03-19

**Authors:** Jang-Hyun Baek, Byung Moon Kim, Ji Hoe Heo, Dong Joon Kim, Hyo Suk Nam, Young Dae Kim

**Affiliations:** ^1^Department of Neurology, Kangbuk Samsung Hospital, Sungkyunkwan University School of Medicine, Seoul, South Korea; ^2^Department of Neurology, Severance Stroke Center, Severance Hospital, Yonsei University College of Medicine, Seoul, South Korea; ^3^Department of Radiology, Interventional Neuroradiology, Severance Stroke Center, Severance Hospital, Yonsei University College of Medicine, Seoul, South Korea

**Keywords:** endovascular treatment, intracranial atherosclerosis, vertebrobasilar occlusion, occlusion type, clinical outcome

## Abstract

**Background and Purpose:** Endovascular treatment (EVT) for acute vertebrobasilar intracranial atherosclerosis-related large vessel occlusion (ICAS-LVO) and its outcomes are not well known. We aimed to evaluate endovascular and clinical outcomes of vertebrobasilar ICAS-LVO patients who underwent EVT.

**Methods:** Consecutive acute stroke patients who underwent EVT for vertebrobasilar LVO were retrospectively reviewed. Patients were assigned to the ICAS (+) or the ICAS (–) group based on angiographical findings. Procedural details and clinical outcomes were compared between the ICAS (+) and ICAS (–) groups.

**Results:** This study included 77 patients with acute vertebrobasilar LVO who underwent EVT. Among the study subjects, 24 (31.2%) had an ICAS-LVO. Recanalization was achieved in 19 patients in the ICAS (+) group (79.2%), which was comparable with the ICAS (–) group (84.9%; *p* = 0.529). However, recanalization using conventional endovascular modalities (stent retriever thrombectomy, contact aspiration thrombectomy, or intra-arterial urokinase infusion) was less successful in the ICAS (+) group (36.8%) than the ICAS (–) group (100.0%; *p* < 0.001). All the remaining patients in the ICAS (+) group required specific rescue treatments appropriate for ICAS, including balloon angioplasty, stenting, or intra-arterial glycoprotein IIb/IIIa inhibitor infusion to obtain a successful recanalization. Procedural time was not significantly longer in the ICAS (+) group. The rates of favorable outcomes (37.5% vs. 41.5%; *p* = 0.740), death, and symptomatic intracerebral hemorrhage were not significantly different between the groups.

**Conclusion:** ICAS-LVO was common in patients who underwent EVT for acute vertebrobasilar LVO. Although conventional modalities were often ineffective for vertebrobasilar ICAS-LVO, a comparable recanalization rate could be obtained with ICAS-specific modalities. Recanalization rate and procedural time were comparable, and clinical outcomes did not differ between patients with or without ICAS-LVO.

## Introduction

Due to the significant improvement of recanalization rate in modern endovascular treatment (EVT) of large vessel occlusion (LVO), we are now focusing on types of intractable cases (1–4). An LVO caused by *in situ* thrombo-occlusion from underlying intracranial atherosclerosis (intracranial atherosclerosis-related LVO, ICAS-LVO) is considered one of the intractable cases. Subsequently, devising an optimal endovascular strategy for management of ICAS-LVO is important. Then, for an optimal endovascular strategy, reliable information is required such as prediction, procedural details, and endovascular and clinical outcomes of ICAS-LVO.

Because ICAS-LVO is more frequent in posterior circulation (5–7), an endovascular strategy for ICAS-LVO might be more important in procedures for vertebrobasilar LVO than LVO in anterior circulation. Moreover, patients with vertebrobasilar LVO have higher morbidity and mortality compared with LVO in anterior circulation (8, 9). Although there are several nonspecific factors affecting the prognosis of acute vertebrobasilar LVO (10), they have not been actively utilized when selecting patients eligible for EVT (11–13). In fact, all recent randomized controlled trials were performed only with LVO in anterior circulation, and substantial criteria were established to minimize futile recanalization of LVO in anterior circulation. In contrast to LVO in anterior circulation, greater focus has been on the recanalization procedure in vertebrobasilar LVO rather than patient selection factors. Therefore, efficient recanalization of ICAS-LVO might be a more important issue especially in vertebrobasilar LVO. In addition, EVT of the vertebrobasilar ICAS-LVO should be understood in the context of patient clinical outcomes.

Information regarding vertebrobasilar ICAS-LVO is lacking. The procedural details and endovascular and clinical outcomes of vertebrobasilar ICAS-LVO have been reported in only a few studies (6, 7). Accordingly, we evaluated the procedural details and clinical outcomes of vertebrobasilar ICAS-LVO patients treated with EVT.

## Methods

We retrospectively reviewed consecutive acute stroke patients who underwent EVT for intracranial LVO in posterior circulation in a tertiary stroke center from September 2010 to June 2018. The intracranial LVO was restricted to occlusion of a basilar artery or intracranial segment of a vertebral artery (vertebrobasilar artery). The Institutional Review Board approved this study and waived the requirement of informed consent for this study due to its retrospective design. For patients eligible for intravenous tissue-type plasminogen activator (tPA) treatment, a full dose (0.9 mg/kg) of tPA was administered. EVT was considered for patients with a computed tomography angiography (CTA)-determined endovascularly accessible LVO relevant to neurological symptoms, initial National Institutes of Health Stroke Scale score ≥ 4, and stroke onset within 12 h.

### Endovascular Treatment

According to the predetermined protocol, a stent retriever (Solitaire; Medtronic, Minneapolis, MN or Trevo; Stryker, Kalamazoo, MI) was used as the first endovascular modality in most procedures. A balloon-guiding catheter was not used in endovascular procedures. All procedures were performed under local anesthesia.

In patients with LVO who did not respond to several trials of stent retriever or who showed significant stenosis on the occlusion segment, rescue EVTs were considered. Endovascular modalities for rescue were contact aspiration thrombectomy with Penumbra Reperfusion Catheter (Penumbra, Alameda, CA) or Sofia (Microvention, Tustin, CA), intra-arterial urokinase infusion, balloon angioplasty, intracranial stenting, and/or intra-arterial glycoprotein IIb/IIIa inhibitor (GPI) infusion. Decision for optimal rescue endovascular modalities was based on the operator's best judgment. In most patients who underwent intracranial stenting, intra-arterial GPI was administered to resolve or prevent in-stent thrombosis. We performed the flat-panel CT before GPI infusion in most cases of patients. Although we did not have a specific criterion of unfavorable condition for GPI infusion, large contrast enhancement was the most common reason not to use GPI or to lessen the total dose of GPI. Typically, 5–10 mg of abciximab (typically 1–2 mg/min) or 0.5–2.0 mg of tirofiban (0.05 mg/ml concentration with 0.1 mg/min) was infused intra-arterially. To secure the stability of arterial patency after balloon angioplasty, intracranial stenting, or intra-arterial GPI infusion, serial delay angiograms were collected for at least 20 min after recanalization. The procedure was finished only when significant angiographic worsening was not observed in arterial patency or perfusion status. Intravenous maintenance of GPI after the EVT was also considered if necessary. Oral dual antiplatelet medication was started from the day after the EVT procedure if there was no significant intracranial hemorrhage or other hemorrhagic complication.

Successful recanalization was defined as a modified Thrombolysis Cerebral Ischemia grade 2b or 3, which should not accompany reocclusion events on delay angiograms. Reocclusion event was defined as a complete or incomplete occlusion event after sufficient recanalization. To identify the reocclusion events, follow-up angiography was performed for at least 20 min after recanalization in all patients.

### Determination of ICAS-LVO

**Figure 1:**
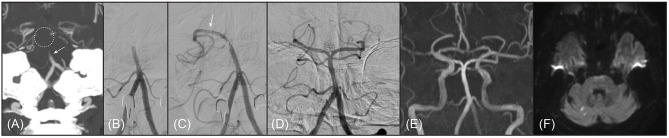
A representative example of 67-year old male patient with branching-site occlusion. **(A,B)** Basilar artery (BA) occlusion was noted on preprocedural computed tomography angiography (**A**, coronal view of maximal intensity projection image; arrow) and digital subtraction angiography (**B**, anteroposterior view). BA bifurcation site was not clearly seen on preprocedural computed tomography angiography (dotted circle). **(C)** Post-deployment angiogram showed only unilateral posterior cerebral artery was observed, which could be considered as a finding of branching-site occlusion (branch-missing sign). Distal marker of the stent retriever was seen on the angiogram (arrow). **(D)** With 1 stent retriever thrombectomy, the BA was completely recanalized. **(E)** On follow-up magnetic resonance angiography (time-of-flight image) performed 1 day after procedure, the recanalized BA was patent. **(F)** Only multiple tiny acute infarctions were noted in bilateral cerebellum and occipital lobe on diffusion-weighted magnetic resonance images. The patient discharged without any neurologic deficits, whose modified Rankin Scale score was 0 at 3 months after stroke.

**Figure 2:**
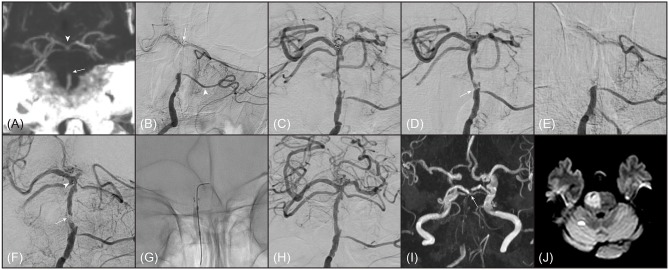
A representative example of 80-year old female patient with truncal-type occlusion. **(A)** On preprocedural computed tomography angiography (coronal view of maximal intensity projection image), occlusion of basilar artery (BA) was noted (arrow). On the computed tomography angiography, BA bifurcation was clearly seen (arrowhead). **(B)** On digital subtraction angiography (anteroposterior view), BA occlusion was also noted. BA bifurcation (arrow) and bilateral posterior cerebral arteries were seen by collateral flows through left anterior inferior cerebellar artery (arrowhead). **(C)** BA bifurcation and bilateral posterior cerebral arteries were definitely verified by stent-through flow. **(D)** By 1 stent retriever thrombectomy, BA occlusion was recanalized, however, focal stenosis with angiographical haziness was noted at the original occlusion site (arrow). **(E)** The recanalized BA was immediately reoccluded. Even with intra-arterial administration of tirofiban 0.5 mg, BA was not recanalized. **(F)** Intracranial stenting with Solitaire (Medtronic, Minneapolis, MN) was performed across the occlusion segment. Distal marker of Solitaire was observed at distal BA (arrowhead). Despite intracranial stenting, arterial patency was not well maintained, which suggested an impending occlusion (arrow). **(G)** Balloon angioplasty with Gateway (Stryker, Kalamazoo, MI) was performed. **(H)** BA was successfully recanalized with mild stenosis and did not show reocclusion event. **(I)** On follow-up magnetic resonance angiography (time-of-flight image) performed 1 day after procedure, distal BA flow was well maintained. (arrow) **(J)** Acute infarction was noted in most of right pons on diffusion-weighted magnetic resonance images. Her modified Rankin Scale score was 5 at 3 months after stroke.

ICAS-LVO was determined based on occlusion type. All occlusions were classified as either branching site or truncal type primarily based on digital subtraction angiography (DSA) findings (14). Briefly, if arterial bifurcation and all its distal branches beyond the occlusion segment were saved, it was considered a truncal-type occlusion. Based on the principal theory, the truncal-type occlusion can be regarded as an ICAS-LVO (Figures 1,2).

The occlusion type was assessed by 2 independent neurointerventionalists. The kappa value for the inter-rater reliability of DSA-determined occlusion type was 0.92. Discrepant cases were decided by consensus among reviewers. For cases whose occlusion type could not be determined based on DSA, mostly due to poor image quality or invalid distal confirmation, the occlusion type was determined using CTA (15). Inter-rater reliability of the CTA-determined occlusion type was excellent (kappa, 0.98).

### Clinical Outcomes

Clinical outcomes included functional outcome, death, and symptomatic intracerebral hemorrhage (SICH). Functional outcome and death were assessed using the modified Rankin Scale (mRS) score at 3 months after stroke onset. Favorable outcome was defined as mRS score 0–2. The functional outcome was primarily evaluated by stroke neurologists during the patient's routine clinic follow-up at 3 months ± 2 weeks. If a patient could not come to the clinic, a stroke neurologist or trained nurse interviewed the patient or their family via telephone to determine the mRS score.

ICH was evaluated on follow-up CT or magnetic resonance (gradient echo) images obtained 24 ± 6 h after EVT. The ICH was finally determined by consensus among stroke neurologists, neurointerventionalists, and neuroradiologists during regular stroke conferences. The determination of ICH was immediately entered into the prospective registry. ICH was regarded as symptomatic if National Institutes of Health Stroke Scale score increased ≥4.

### Statistical Analysis

Based on the occlusion type determined, patients were assigned to either the ICAS (+) group or the ICAS (–) group. Patients with a truncal-type occlusion were assigned to the ICAS (+) group. Demographics, common risk factors for stroke, procedural details and outcomes, and clinical outcomes were compared between ICAS (+) and ICAS (–) groups. Mann-Whitney *U* test, χ^2^ test, and Fisher exact test were used for comparison. Also, multivariable logistic regression analysis was also performed to independent factors for favorable outcome. For this analysis, age, variables with *P*-value < 0.1, time profiles including onset-to-puncture and puncture-to-recanalization time, and ICAS-LVO were adjusted. A *P*-value < 0.05 was considered statistically significant with a 95% confidence interval (CI). Statistical analyses were performed using software (version 3.4.2; r-project.org).

## Results

**Figure 3:**
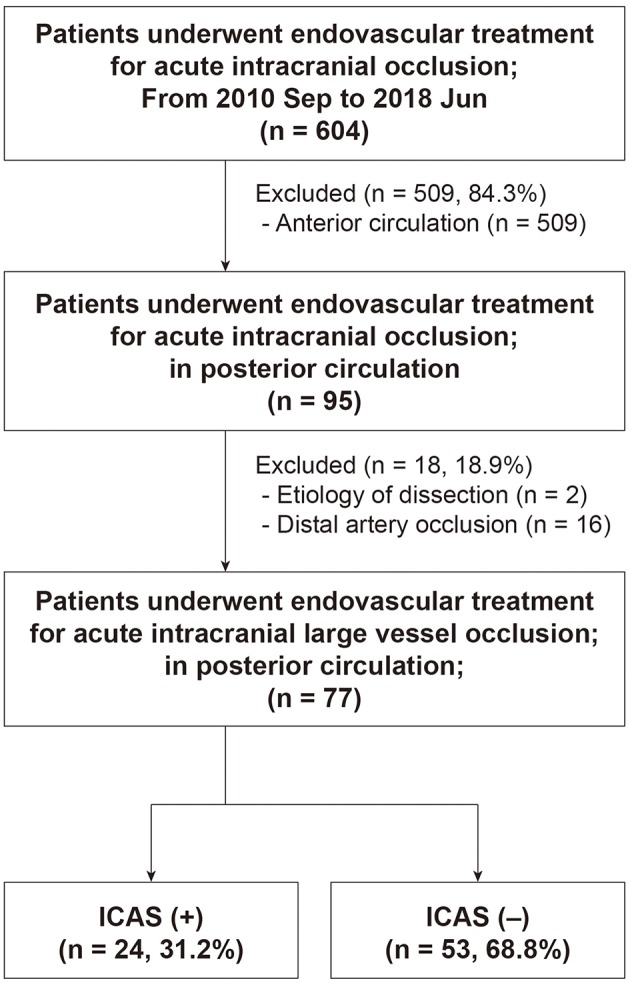
Flow chart of patient selection. ICAS indicates intracranial atherosclerosis.

Among 604 patients who underwent EVT for an intracranial LVO, 77 (mean age, 73.2 ± 12.8 years; male, 53.2%) were finally included (Figure 3). Patients with distal artery occlusion (*n* = 16) and etiology of arterial dissection (*n* = 2) were also excluded. In the study population, 69 patients (89.6%) had a basilar artery occlusion and 8 (10.4%) had an intracranial vertebral artery occlusion. Occlusion type was determined based on DSA in 67 patients (87.0%) and CTA in 10 patients (13.0%). Among the included patients, 24 (31.2%) had an ICAS-LVO. Atrial fibrillation was less frequent in the ICAS (+) group, whereas intravenous tPA was more frequent in the ICAS (+) group (Table 1). Stroke severity was not significantly different between groups—median initial NIHSS score in the ICAS (+) group was 14.5 and 12.0 in the ICAS (–) group (*P* = 0.624; Supplemental Figure 1).

**Table 1:** Comparison of variables between patients with and without vertebrobasilar intracranial atherosclerosis-related acute large vessel occlusion (ICAS-LVO).

	**Total patients (*n* = 77)**	**ICAS (+) (*n* = 24)**	**ICAS (–) (*n* = 53)**	***P*-value**	Odds ratio^*^ (95% CI)
Age, years	73.2 (±12.8)	72.7 (±13.8)	73.5 (±12.5)	0.800	0.99 (0.96–1.03)
Sex, male	41 (53.2)	14 (58.3)	27 (50.9)	0.547	1.34 (0.51–3.57)
Hypertension	62 (80.5)	21 (87.5)	41 (77.4)	0.366	2.05 (0.52–8.06)
Diabetes	30 (39.0)	6 (25.0)	24 (45.3)	0.091	0.40 (0.14–1.17)
Dyslipidemia	19 (24.7)	6 (25.0)	13 (24.5)	0.965	1.03 (0.34–3.13)
Smoking	10 (13.0)	7 (29.2)	3 (5.7)	0.008	6.86 (1.59–29.6)
Coronary artery disease	24 (31.2)	5 (20.8)	19 (35.8)	0.188	0.47 (0.15–1.46)
Atrial fibrillation	36 (46.8)	7 (29.2)	29 (54.7)	0.037	0.34 (0.12–0.96)
Initial NIHSS score	13.0 [7.0; 24.0]	14.5 [9.0; 21.5]	12.0 [7.0; 25.0]	0.624	1.00 (0.95–1.05)
Basilar artery occlusion	69 (89.6)	16 (66.7)	53 (100.0)	<0.001	N/A
Use of IV tPA	18 (23.4)	10 (41.7)	8 (15.1)	0.011	4.02 (1.33–12.1)
Onset-to-puncture, min	262.0 [146.0; 435.0]	207.0 [158.0; 307.8]	285.0 [140.0; 475.0]	0.253	0.94 (0.87–1.02)^†^

*CI, confidence interval; NIHSS, National Institutes of Health Stroke Scale; IV tPA, intravenous tissue plasminogen activator; N/A, not applicable*.

*Values in parentheses represent the standard deviation, number of patients (%), or median; brackets represent first and third quartiles*.

* Odds ratio for ICAS (+)

† *Odds ratio per 30 min of time*.

### Procedural Details and Outcomes

Successful recanalization was achieved in 64 patients (83.1%). Successful recanalization rate in the ICAS (+) group was similar to that in the ICAS (–) group (79.2% vs. 84.9%; *P* = 0.529; Table 2). All patients in the ICAS (–) group obtained a successful recanalization with conventional endovascular modalities, of which most (93.3%) were mechanical thrombectomy devices. Conversely, only 36.8% of patients in the ICAS (+) group achieved a successful recanalization with conventional endovascular modalities (*P* < 0.001). The remaining patients in the ICAS (+) group (63.2%; *n* = 12) eventually required ICAS-specific modalities to achieve a successful recanalization, including intra-arterial GPI infusion, balloon angioplasty, and intracranial stenting. Among the 12 patients treated with ICAS-specific modalities, 8 obtained a successful recanalization only with GPI infusion (66.7%), and 3 (25.0%) eventually required rescue stenting. For intracranial stenting, Solitaire was used in all cases. In patients in the ICAS (+) group treated with GPI (*n* = 11), abciximab was infused in 6 (54.5%; mean dose, 9.2 ± 3.4 mg) and tirofiban in 5 patients (45.5%; 0.7 ± 0.2 mg).

**Table 2:** Procedural and clinical outcomes of patients with and without vertebrobasilar intracranial atherosclerosis-related acute large vessel occlusion (ICAS-LVO).

	**Total patients (*n* = 77)**	**ICAS (+) (*n* = 24)**	**ICAS (–) (*n* = 53)**	***P*-value**	Odds ratio^*^ (95% CI)
**ENDOVASCULAR OUTCOMES**					
Successful recanalization	64 (83.1)	19 (79.2)	45 (84.9)	0.529	0.68 (0.20–2.33)
Conventional modalities	52 (81.2)	7 (36.8)	45 (100.0)	<0.001	N/A
Stent retriever	38 (59.4)	5 (26.2)	33 (73.3)		
Contact aspiration thrombectomy	10 (15.6)	1 (5.3)	9 (20.0)		
Urokinase	4 (6.2)	1 (5.3)	3 (6.7)		
ICAS-specific modalities	12 (18.8)	12 (63.2)	0 (0.0)		
GPI	8 (12.5)	8 (42.1)	0 (0.0)		
PTA	1 (1.6)	1 (5.3)	0 (0.0)		
Stenting + PTA + GPI	3 (4.7)	3 (15.8)	0 (0.0)		
Procedural Events					
Reocclusion during the procedure	18 (23.4)	15 (62.5)	3 (5.7)	<0.001	27.8 (6.66–115.9)
Puncture-to-recanalization, min	46.5 [27.8; 93.2]	52.0 [25.5; 117.5]	45.0 [28.0; 77.0]	0.837	1.07 (0.80–1.44)^†^
Onset-to-recanalization, min	351.0 [207.5; 497.2]	325.0 [243.0; 437.5]	383.0 [201.0; 529.0]	0.791	0.97 (0.90–1.06)^†^
**CLINICAL OUTCOMES**					
Favorable outcome	31 (40.3)	9 (37.5)	22 (41.5)	0.740	0.85 (0.32–2.28)
Death	16 (20.8)	4 (16.7)	12 (22.6)	0.763	0.68 (0.20–2.39)
Symptomatic ICH	4 (5.2)	3 (12.5)	1 (1.9)	0.087	7.43 (0.73–75.5)

*CI, confidence interval; GPI, glycoprotein IIb/IIIa inhibitor; PTA, percutaneous transluminal angioplasty; ICH, intracerebral hemorrhage; N/A, not applicable*.

*Values in parentheses represent the number of patients (%) or median; brackets represent first and third quartiles*.

* *Odds ratio for ICAS (+)*.

† *Odds ratio per 30 min of time*.

Reocclusion events after initial recanalization using conventional endovascular modalities were observed more frequently in the ICAS (+) group than in the ICAS (–) group (62.5% vs. 5.7%; *P* < 0.001; Table 2). In 15 patients with reocclusion events in the ICAS (+) group, GPI was used in 13 (86.7%). Reocclusion events were resolved only with GPI infusion in 8 patients (61.5%; 8 of 13). For patients in whom GPI infusion was unsuccessful, additional consecutive intracranial stenting with balloon angioplasty provided more recanalization in 3 patients (23.1%; 3 of 13). Altogether, 84.6% (11 of 13) of patients with reocclusion events were successfully recanalized with GPI infusion and/or consecutive intracranial stenting with balloon angioplasty.

Median puncture-to-recanalization time was 46.5 min (interquartile range, 27.8–93.2; range, 9.0–273.0; Supplemental Figure 2) and median onset-to-recanalization time was 351.0 min (interquartile range, 207.5–497.2; range, 99.0–1020.0; Supplemental Figure 3). Puncture-to-recanalization time was not significantly different between the ICAS (+) and the ICAS (–) groups (52.0 vs. 45.0 min; *P* = 0.837). Onset-to-recanalization time was not significantly different between the groups, either (325.0 vs. 383.0 min; *P* = 0.791).

### Clinical Outcomes

**Figure 4:**
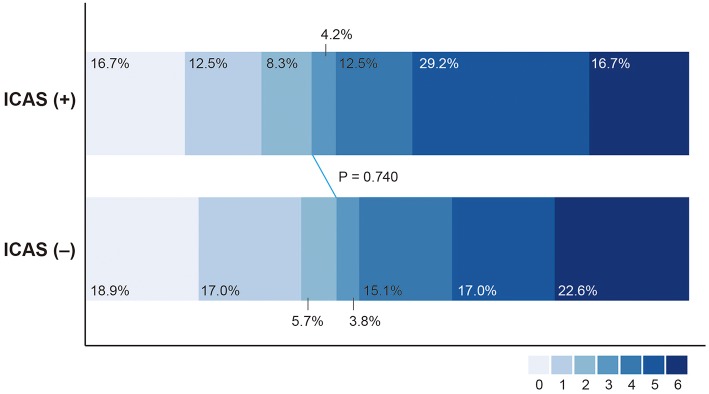
Distribution of modified Rankin Scale score at 3 months after treatment in patients with intracranial atherosclerosis-related large vessel occlusion (ICAS [+]) and without (ICAS [–]).

Favorable outcome (37.5% vs. 41.5%; *P* = 0.740) and death (16.7% vs. 22.6%; *P* = 0.763) were not significantly different between the ICAS (+) and ICAS (–) groups (Table 2, Figure 4). SICH developed in 4 patients (5.2%) in the study population. SICH was higher in the ICAS (+) group than the ICAS (–) group; however, the difference was not statistically significant (12.5% vs. 1.9%; *P* = 0.087). SICH developed in 12.5% of patients who underwent intra-arterial GPI infusion, which was not significantly higher than in patients who did not receive intra-arterial GPI infusion (3.3%; *P* = 0.189). In addition, use of intravenous tPA was not associated with development of SICH (*P* = 0.999). For patients with basilar artery occlusion (*n* = 69), clinical outcomes were not significantly different between the ICAS (+) and ICAS (–) groups (Supplemental Table).

In multivariable analysis, initial NIHSS score (odds ratio 0.82 with 95% confidence interval 0.74–0.91; *P* < 0.001; Table 3) and puncture-to-recanalization time (odds ratio 0.81 per 10 min with 0.65–0.99; *P* = 0.046) were independent factors for favorable outcome.

**Table 3:** Univariable and multivariable analyses of variables affecting favorable outcome.

	**Univariable analysis**			**Multivariable analysis**	
	**Favorable outcome (*n* = 31)**	**Unfavorable outcome (*n* = 46)**	***P*-value**	**Odds ratio (95% CI)**	***P*-value**
Age, years	70.9 (±15.3)	74.8 (±10.8)	0.216	0.97 (0.92–1.04)	0.397
Sex, male	18 (58.1)	23 (50.0)	0.487		
Hypertension	24 (77.4)	38 (82.6)	0.573		
Diabetes	10 (32.3)	20 (43.5)	0.322		
Dyslipidemia	6 (19.4)	13 (28.3)	0.374		
Smoking	5 (16.1)	5 (10.9)	0.512		
Coronary artery disease	9 (29.0)	15 (32.6)	0.740		
Atrial fibrillation	17 (54.8)	19 (41.3)	0.243		
Initial NIHSS score	7.0 [5.5; 11.5]	22.0 [12.2; 28.0]	<0.001	0.82 (0.74–0.91)	<0.001
Basilar artery occlusion	29 (93.5)	40 (87.0)	0.463		
Use of IV tPA	8 (25.8)	10 (21.7)	0.679		
Onset-to-puncture, min	210.0 [123.0; 415.5]	265.5 [169.2; 440.2]	0.347	0.96 (0.86–1.06)^*^	0.414
Puncture-to-recanalization, min	35.0	56.0	0.001	0.81	0.046
	[19.0; 58.0]	[38.0; 130.5]		(0.65–0.99)^†^	
Onset-to-recanalization, min	264.0 [182.0; 443.0]	390.0 [294.0; 497.5]	0.180		
Successful recanalization	29 (93.5)	35 (76.1)	0.063		
Reocclusion during the procedure	4 (12.9)	14 (30.4)	0.075	0.43 (0.04–5.07)	0.504
Use of GPI during procedure	5 (16.1)	11 (23.9)	0.409		
Symptomatic ICH	0 (0.0)	4 (8.7)	0.143		
ICAS-related large vessel occlusion	9 (29.0)	15 (32.6)	0.740	1.10 (0.15–8.21)	0.924

*CI, confidence interval; NIHSS, National Institutes of Health Stroke Scale; IV tPA, intravenous tissue plasminogen activator; GPI, glycoprotein IIb/IIIa inhibitor; ICH, intracerebral hemorrhage; ICAS, intracranial atherosclerosis*.

*Values in parentheses represent the standard deviation, number of patients (%), or median; brackets represent first and third quartiles*.

* *Odds ratio per 30 min of time*.

† *Odds ratio per 10 min of time*.

## Discussion

In this study, ICAS-LVO comprised approximately 31% of all vertebrobasilar LVOs eligible for EVT. Patients with ICAS-LVO required specific rescue treatments appropriate for underlying ICAS, which resulted in a successful recanalization rate similar to that of patients without ICAS-LVO, and clinical outcomes in patients with or without ICAS-LVO were not significantly different.

ICAS-LVO is more frequent in patients with acute vertebrobasilar occlusion eligible for EVT (6, 7). Kim et al. reported that approximately 37% of ICAS-LVO in acute vertebrobasilar artery occlusion has been observed in the Korean population (6). The reported frequency of ICAS-LVO did not differ from our study. Lee et al. also reported the frequency of ICAS-LVO in acute basilar artery occlusion as approximately 24%, similar to our study (23.2%; 16 of 69 basilar artery occlusions) (7). Because most ICAS-LVO studies included LVOs in both anterior and posterior circulation, comparing frequencies of ICAS-LVO between anterior and posterior circulation is difficult (5, 16–18). The frequency of ICAS-LVO in both circulations was approximately 8–23%, far less than posterior circulation studies (6, 7). In addition, posterior circulation involvement was an independent factor associated with ICAS-LVO (5). The reason ICAS-LVO is more prevalent in posterior circulation is unclear. However, an optimal endovascular strategy for ICAS-LVO might be more important in posterior than anterior circulation cases due to higher frequency.

Clinical outcomes of patients with vertebrobasilar ICAS-LVO who underwent EVT were not consistent in previous studies. Lee et al. showed that patients with vertebrobasilar ICAS-LVO had comparable clinical outcomes to those without vertebrobasilar ICAS-LVO (7). Conversely, in another study, patients with vertebrobasilar ICAS-LVO had a less favorable outcome despite similar recanalization rates (6). Furthermore, vertebrobasilar ICAS-LVO was an independent factor for poor prognosis. One potential reason for the difference of clinical outcome was procedural time. Based on those studies, longer procedural time was associated with poor clinical outcomes (7). In this study, time profiles including onset-to-recanalization time and puncture-to-recanalization time were not significantly longer in patients with vertebrobasilar ICAS-LVO. In addition, clinical outcomes were also comparable to those of patients without vertebrobasilar ICAS-LVO. However, even in this study, puncture-to-recanalization time was an independent factor for favorable outcome. Considering the findings from previous and current studies, procedural time could be an important factor that influences clinical outcome in acute vertebrobasilar ICAS-LVO. Conversely, recanalization success might be an important factor affecting clinical outcomes of patients with ICAS-LVO of anterior circulation because their clinical outcomes were proportional to successful recanalization rate (16, 18–20).

Occlusion location might affect the stroke severity in LVO of posterior circulation. In BA occlusion, patients with proximal or middle clot had higher mortality than distal (21). Clinical outcome was not favorable in proximal or middle clot, either. Another study also showed a higher mortality in BA occlusion with atherothrombosis, which were represented as a proximal and middle BA occlusion (22). One possible explanation for the more severe stroke severity in the ICAS-LVO group is the involvement of pons perforator. In our study, an initial NIHSS score was higher in the ICAS-LVO group. This might be come from that branching-site occlusion absolutely involves distal BA based on its original theory, while the truncal-type occlusion might be possible at all segments of BA including its proximal and middle part.

Procedural details to treat vertebrobasilar ICAS-LVO are not well known. The feasibility of intracranial stenting, balloon angioplasty, and intra-arterial GPI infusion, termed ICAS-specific endovascular modalities in vertebrobasilar ICAS-LVO, have been reported only in a few case series (23, 24). Kim et al. only reported the use of ICAS-specific modalities based on the presence of vertebrobasilar ICAS-LVO (6). More ICAS-specific modalities were used in patients with vertebrobasilar ICAS-LVO for rescue treatment. However, the study did not offer detailed information regarding the success rate of modern conventional modalities (e.g., stent retriever or contact aspiration thrombectomy) or effectiveness of each ICAS-specific modality in vertebrobasilar ICAS-LVO. Procedural details of the current study were similar to those of other studies on ICAS-LVO (18, 19, 23–26). To obtain a successful recanalization, ICAS-LVO required significantly more rescue treatments, which were all ICAS-specific modalities. Apparently, ICAS-specific modalities were feasible and necessary in most patients with vertebrobasilar ICAS-LVO. Interestingly, among them, intra-arterial GPI infusion could be considered as the first ICAS-specific modality. In this study, 66.7% of patients who were treated by ICAS-specific modalities could get a successful recanalization by intra-arterial GPI alone. In anterior circulation ICAS-LVOs, intra-arterial GPI was effective in about 40% of patients without the use of other ICAS-specific modalities (20). According to the response to the first intra-arterial GPI infusion, one might consider intracranial stenting or balloon angioplasty.

In summary, the expected successful recanalization rate in vertebrobasilar ICAS-LVO using modern conventional modalities is not high, possibly less than 40%. In addition, ICAS-specific endovascular modalities were quite feasible and effective. Furthermore, shorter procedural time appears more important in vertebrobasilar ICAS-LVO for better clinical outcome. Rapid and active introduction of ICAS-specific modalities should be considered as an optimal endovascular strategy in vertebrobasilar ICAS-LVO.

SICH was not statistically different between patients with or without ICAS-LVO; however, frequency of SICH in the ICAS (+) group was higher than in the ICAS (–) group. Most likely, the number of SICH cases was too small to reach statistical significance in this study. Although the number of cases was too small, none were associated with development of SICH among variables used in this study, including use of intra-arterial GPI or intravenous tPA. Further studies are necessary to verify this issue.

This study had several strengths and limitations. First, this study was retrospective; thus, types and specific timing of introduction of rescue endovascular modalities were not protocolized. Although treatment protocol was fundamentally predetermined, many stages during the endovascular procedures were dependent on operator discretion. However, because a reliable method to identify and treatment protocol to manage ICAS-LVO do not exist, the results from this retrospective study might help in the understanding of procedural details for managing vertebrobasilar ICAS-LVO and devising an optimal treatment protocol.

Second, occlusion type was used to identify ICAS-LVO. This is the first study of vertebrobasilar ICAS-LVO that used occlusion type for its identification. Occlusion type is considered a reliable angiographical surrogate marker for ICAS-LVO during or before an endovascular procedure (14, 15). Occlusion type could be determined even in cases with persistent occlusion and had excellent inter-rater reliability. More importantly, endovascular strategy, which is devised from endovascular and clinical results based on the occlusion type, might also be practical for daily EVT procedure. In our center, we actively use occlusion type in identifying the ICAS-LVO in real practice. It might contribute to the comparable procedural time for vertebrobasilar ICAS-LVOs in this study, although it should be verified prospectively. In spite of the clinical advantages, occlusion type might be imperfect in identifying occlusion etiology in some situations. For example, an occlusion by a large size of embolus might be erroneously classified as a truncal-type occlusion. Although occlusion types in this study were mostly determined by DSA, CTA-determined occlusion type considerably depends on collateral adequacy (15). Thus, it seems necessary to compare the occlusion type with another angiographical definition of ICAS-LVO for more reliability (1, 5).

Third, results from this study were derived from a single stroke center in Asia, where ICAS is more prevalent. Thus, generalizability might be limited to a specific population. However, management of ICAS-LVO is challenging in modern EVT, and its importance has been increasing in intractable cases irrespective of ethnicity. Furthermore, in situations where only minimal information regarding procedural and clinical outcomes is available, the current study provides important baseline data for vertebrobasilar ICAS-LVO.

## Conclusions

ICAS-LVO was common in patients who underwent EVT for acute vertebrobasilar LVO. Patients with a vertebrobasilar ICAS-LVO achieved a comparably successful recanalization rate with similar procedural time; however, those patients required more rescue endovascular modalities specific to ICAS-LVO for successful recanalization. The clinical outcomes were comparable in patients with or without vertebrobasilar ICAS-LVO.

## Data Availability

The datasets generated for this study are available on request to the corresponding author.

## Author Contributions

J-HB established the study idea, designed the manuscript structure, acquired and analyzed the data, and wrote the manuscript. BMK established the study idea, designed the manuscript structure, acquired data, and made critical revisions to the manuscript with substantive intellectual content. DJK, JHH, HSN, and YDK acquired data and made critical revisions to the manuscript.

### Conflict of Interest Statement

The authors declare that the research was conducted in the absence of any commercial or financial relationships that could be construed as a potential conflict of interest.
