# Supplementary material for: Endovascular and Clinical Outcomes of Vertebrobasilar Intracranial Atherosclerosis-Related Large Vessel Occlusion
Source: Front Neurol. 2019 Mar 19;10:215. doi: 10.3389/fneur.2019.00215 (PMC6433872; doi:10.3389/fneur.2019.00215)
Supplement: Supplementary file 1 [file Data_Sheet_1.PDF]

Supplemental Table

Supplemental table. Comparison of variables between patients with and without vertebrobasilar intracranial atherosclerosis-related acute large vessel occlusion (ICAS-LVO) of basilar artery

	Total patients (n = 69)	ICAS (+) (n = 16)	ICAS (–) (n = 53)	P-value	Odds ratio* (95% CI)
**Demographics and risk factors**					
Age, years	73.5 (±12.3)	73.6 (±11.8)	73.5 (±12.5)	0.988	1.00 (0.96–1.05)
Sex, male	35 (50.7)	8 (50.0)	27 (50.9)	0.947	0.96 (0.31–2.95)
Hypertension	55 (79.7)	14 (87.5)	41 (77.4)	0.494	2.05 (0.41–10.3)
Diabetes	30 (43.5)	6 (37.5)	24 (45.3)	0.582	0.73 (0.23–2.28)
Dyslipidemia	16 (23.2)	3 (18.8)	13 (24.5)	0.746	0.71 (0.17–2.89)
Smoking	8 (11.6)	5 (31.2)	3 (5.7)	0.014	7.58 (1.57–36.5)
Coronary artery disease	23 (33.3)	4 (25.0)	19 (35.8)	0.420	0.60 (0.17–2.11)
Atrial fibrillation	35 (50.7)	6 (37.5)	29 (54.7)	0.227	0.50 (0.16–1.56)
Initial NIHSS score	13.0 [7.0; 24.0]	14.0 [9.0; 20.2]	12.0 [7.0; 25.0]	0.949	0.99 (0.93–1.05)
Use of IV tPA	14 (20.3)	6 (37.5)	8 (15.1)	0.075	3.38 (0.96–11.9)
Onset-to-puncture, min	270.0 [149.0; 442.0]	264.0 [175.5; 336.5]	285.0 [140.0; 475.0]	0.599	0.96 (0.88–1.05)†
**Endovascular outcomes**					
Successful recanalization	56 (81.2)	11 (68.8)	45 (84.9)	0.162	0.39 (0.11–1.43)
Conventional modalities	50 (89.3)	5 (45.5)	45 (100.0)	<0.001	N/A
Stent retriever	37 (66.1)	4 (36.4)	33 (73.3)		
Contact aspiration thrombectomy	9 (16.1)	0 (0.0)	9 (20.0)		
Urokinase	4 (7.1)	1 (9.1)	3 (6.7)		
ICAS-specific modalities	6 (10.7)	6 (54.5)	0 (0.0)		
GPI	5 (8.9)	5 (45.4)	0 (0.0)		
Stenting + PTA + GPI	1 (1.8)	1 (9.1)	0 (0.0)		
Procedural events					
Reocclusion during the procedure	12 (17.4)	9 (56.2)	3 (5.7)	<0.001	21.4 (4.65–98.7)
Puncture-to-recanalization, min	45 [27.0; 80.5]	42.0 [20.5; 75.0]	45.0 [28.0; 77.0]	0.578	0.90 (0.58–1.40)†
Onset-to-recanalization, min	386.5 [214.2; 505.8]	405.0 [316.5; 468.5]	383.0 [201.0; 529.0]	0.650	1.00 (0.91–1.10)†
**Clinical outcomes**					
Favorable outcome	27 (42.0)	7 (43.8)	22 (41.5)	0.874	1.10 (0.35–3.39)
Death	12 (17.4)	0 (0.0)	12 (22.6)	0.055	N/A
Symptomatic ICH	3 (4.3)	2 (12.5)	1 (1.9)	0.132	7.43 (0.63–87.9)

CI indicates confidence interval; NIHSS, National Institutes of Health Stroke Scale; IV tPA, intravenous tissue plasminogen activator; GPI, glycoprotein IIb/IIIa inhibitor; PTA, percutaneous transluminal angioplasty; ICH, intracerebral hemorrhage; N/A, not applicable.

Values in parentheses represent the standard deviation, the number of patients (%), or median; brackets represent first and third quartiles.

*Odds ratio for ICAS (+)

†Odds ratio per 30 minutes of time

Supplemental Figures

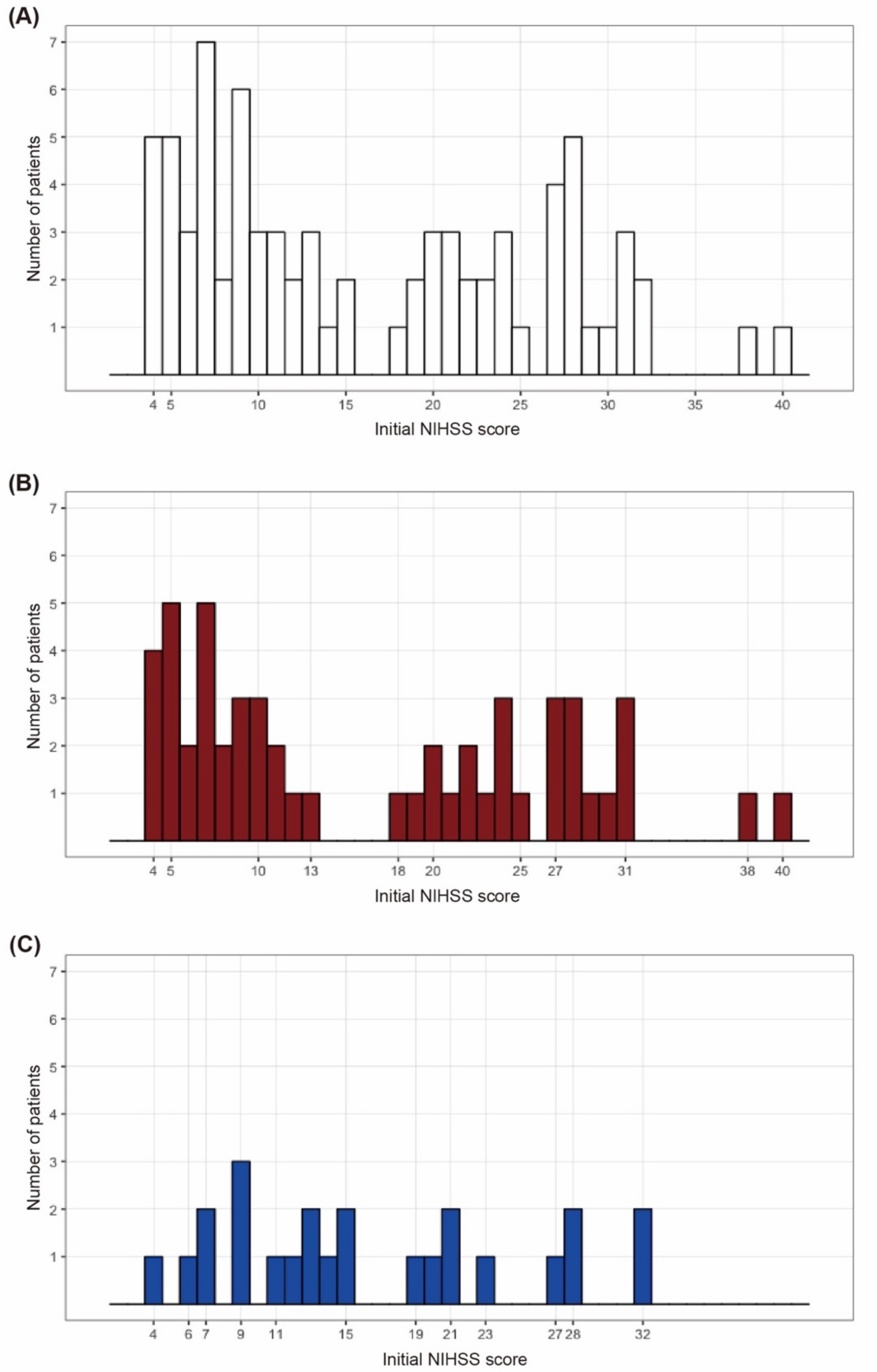

Supplemental figure 1. Distribution of initial National Institutes of Health Stroke Scale (NIHSS) score (A) All patients (n = 77; median 14.5 with interquartile range [IQR] 7.0 –24.0; range 4 – 40). **(B)** Patients without intracranial atherosclerosis-related large vessel occlusion (ICAS-LVO) (n = 53; median 12.0 with IQR 7.0 –25.0; range 4 – 40). **(C)** Patients with ICAS-LVO (n = 24; median 14.5 with IQR 9.0 – 21.5; range 4 – 32).

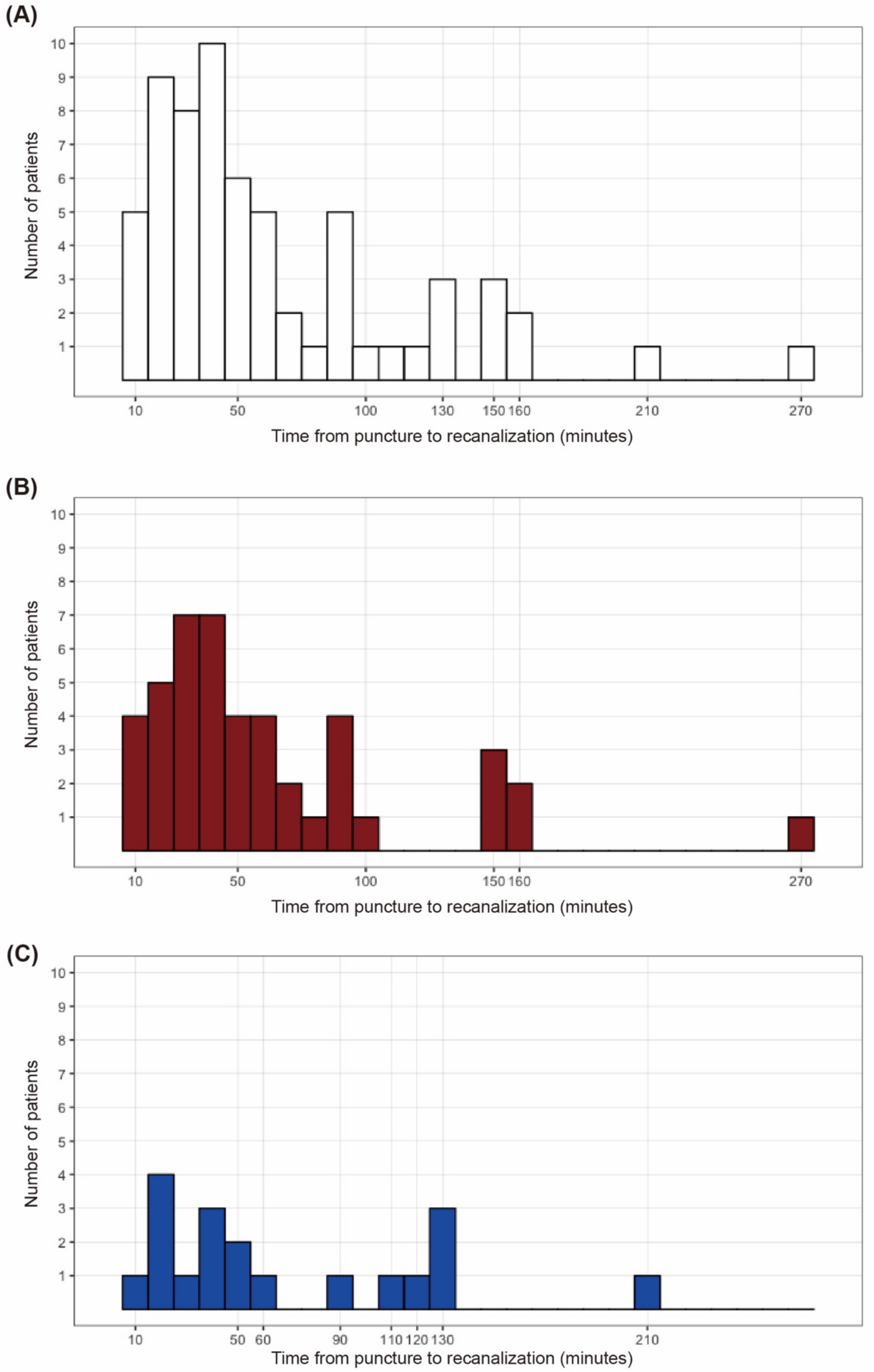

Supplemental figure 2. Distribution of puncture-to-recanalization time (A) All patients (n = 64; median 46.5 minutes with interquartile range [IQR] 27.8 – 93.2; range, 9.0 – 273.0). **(B)** Patients without intracranial atherosclerosis-related large vessel occlusion (ICAS-LVO) (n = 45; median 45.0 with IQR 28.0 – 77.0; range 9.0 – 273.0). **(C)** Patients with ICAS-LVO (n = 19; median 52.0 with IQR 25.5 – 117.5; range, 14.0 – 207.0).

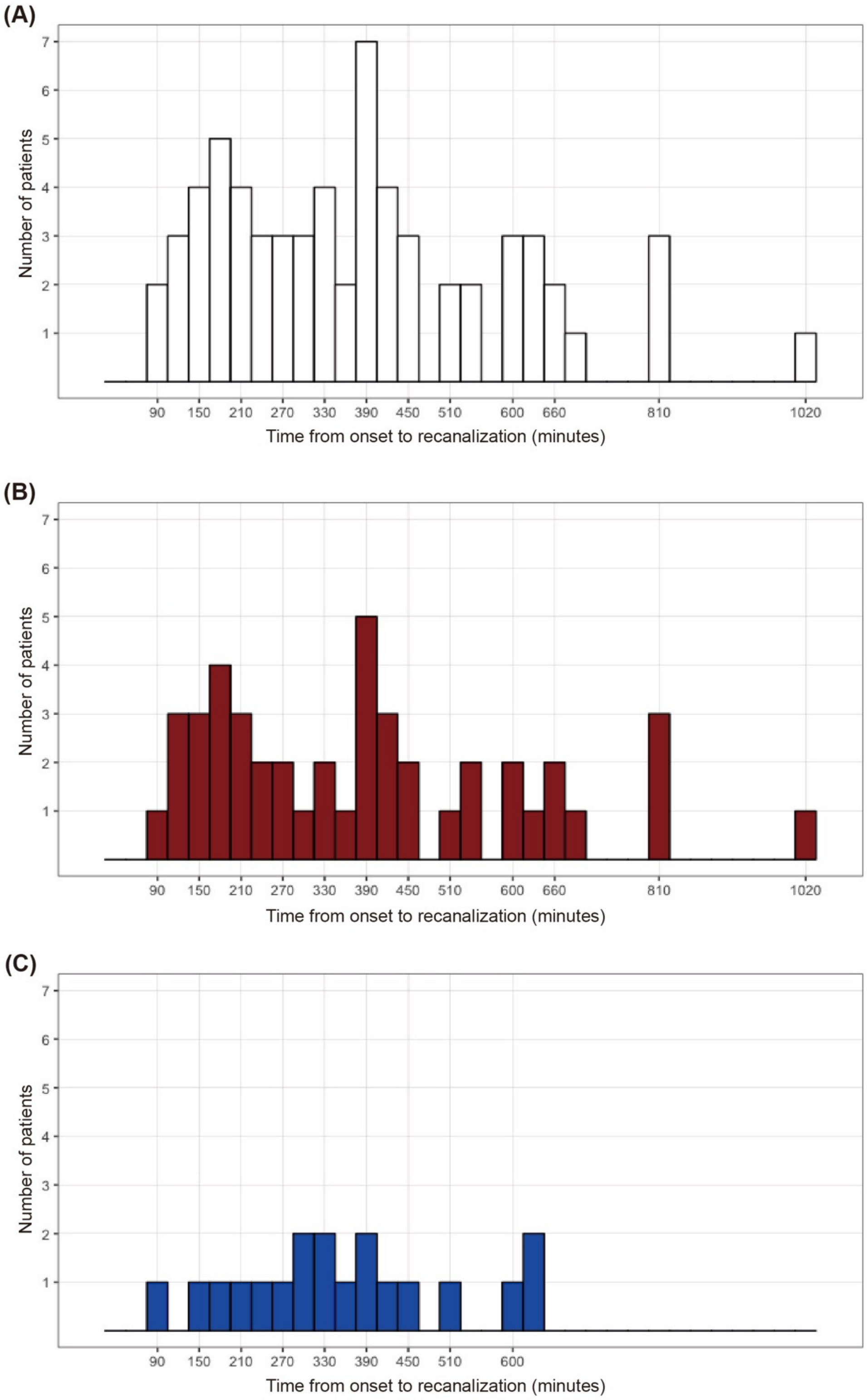

Supplemental figure 3. Distribution of onset-to-recanalization time (A) All patients (n = 64; median 351.0 minutes with interquartile range [IQR] 207.5 – 497.2; range, 99.0 – 1020.0). **(B)** Patients without intracranial atherosclerosis-related large vessel occlusion (ICAS-LVO) (n = 45; median 383.0 with IQR 201.0 – 392.2; range 101.0 – 1020.0). **(C)** Patients with ICAS-LVO (n = 19; median 325.0 with IQR 243.0 – 437.5; range, 99.0 – 638.0).
